# Insights into the Early Epidemic Spread of Ebola in Sierra Leone Provided by Viral Sequence Data

**DOI:** 10.1371/currents.outbreaks.02bc6d927ecee7bbd33532ec8ba6a25f

**Published:** 2014-10-06

**Authors:** Tanja Stadler, Denise Kühnert, David A. Rasmussen, Louis du Plessis

**Affiliations:** Department of Biosystems Science and Engineering, ETH Zurich, Basel, Switzerland; Department of Environmental Systems Science, ETH Zurich, Zurich, Switzerland; Department of Biosystems Science and Engineering, ETH Zurich, Basel, Switzerland; Department of Biosystems Science and Engineering, ETH Zurich, Basel, Switzerland

**Keywords:** birth-death, coalescent, ebola, EBOV, phylodynamics

## Abstract

Background and Methodology: The current Ebola virus epidemic in West Africa has been spreading at least since December 2013. The first confirmed case of Ebola virus in Sierra Leone was identified on May 25. Based on viral genetic sequencing data from 72 individuals in Sierra Leone collected between the end of May and mid June, we utilize a range of phylodynamic methods to estimate the basic reproductive number (R0). We additionally estimate the expected lengths of the incubation and infectious periods of the virus. Finally, we use phylogenetic trees to examine the role played by population structure in the epidemic. Results: The median estimates of R0 based on sequencing data alone range between 1.65-2.18, with the most plausible model yielding a median R0 of 2.18 (95% HPD 1.24-3.55). Importantly, our results indicate that, at least until mid June, relief efforts in Sierra Leone were ineffective at lowering the effective reproductive number of the virus. We estimate the expected length of the infectious period to be 2.58 days (median; 95% HPD 1.24-6.98). The dataset appears to be too small in order to estimate the incubation period with high certainty (median expected incubation period 4.92 days; 95% HPD 2.11-23.20). While our estimates of the duration of infection tend to be smaller than previously reported, phylodynamic analyses support a previous estimate that 70% of cases were observed and included in the present dataset. The dataset is too small to show a particular population structure with high significance, however our preliminary analyses suggest that half the population is spreading the virus with an R0 well above 2, while the other half of the population is spreading with an R0 below 1. Conclusions: Overall we show that sequencing data can robustly infer key epidemiological parameters. Such estimates inform public health officials and help to coordinate effective public health efforts. Thus having more sequencing data available for the ongoing Ebola virus epidemic and at the start of new outbreaks will foster a quick understanding of the dynamics of the pathogen.

## Introduction

The 2014 West African Ebola virus (EBOV) epidemic is the largest Ebola virus outbreak to date with 7492 cases (4108 confirmed) and 3439 deaths (2078 confirmed) as of 3 October 2014^1^. While previous EBOV outbreaks remained localized, the current epidemic has spread across Guinea, Sierra Leone and Liberia with a localized outbreak in Nigeria. (Both Senegal and the USA have reported one imported case with no local transmission, as of 3 October 2014). Relief efforts have so far been ineffective at containing the disease, due largely to porous borders, a lack of education about the disease and degraded public health infrastructure^2^^,^ 3 ^,^ 4. Moreover, the epidemic has spread to major urban areas, further facilitating its continued spread and complicating containment efforts.

Patients exposed to EBOV first undergo an incubation period of 2-21 days before becoming infectious^3^^,^ 5 ^,^ 6 ^,^ 7. Once infectious, patients either die between days 6 and 16 or may begin to recover between days 6 and 11^3^^,^ 8 ^,^ 9. Although patients who recover are generally noninfectious after convalescence, EBOV has been isolated 33 days after the onset of symptoms from mucosal membranes and 61 days after the onset of symptoms from semen^10^^,^ 11. There is currently no known effective treatment or vaccine for Ebola virus disease and relief efforts focus on bringing down the case fatality rate through supportive care and disease containment^3^.

In Gire *et al.* (2014)^12^, 99 Ebola genomes from 78 patients from the Sierra Leone outbreak are provided. This represents about 70% of confirmed cases during late May to mid June. Based on the phylogenies in Gire *et al. *(2014)^12^, it is likely that the Sierra Leone outbreak was started by the simultaneous introduction of two genetically distinct viruses. The initial 14 confirmed cases in Sierra Leone have all been epidemiologically linked to the funeral of a traditional healer in Guinea, supporting a single introduction event. The first split of the Sierra Leone sequences, separating the two introductions, is supported in all posterior trees presented in Gire *et al.* (2014)^12^ as well as in our preliminary analyses. We focused on the introduction causing the larger outbreak (72 sampled patients) and ignored the smaller outbreak (6 sampled patients).

We use these genomic data to estimate epidemiological parameters. We employed the Bayesian MCMC framework BEAST2^13^ , applying a range of epidemiological tree priors to the sequencing data. The tree priors are based both on birth-death^14^ and coalescent^15^ models. Furthermore, we estimated epidemiological parameters based on the trees from Gire *et al.* (2014)^12^ using a maximum likelihood framework implemented in R^16^ .

## Methods for epidemiological parameter inference

**The epidemiological models used for phylodynamic inference.**
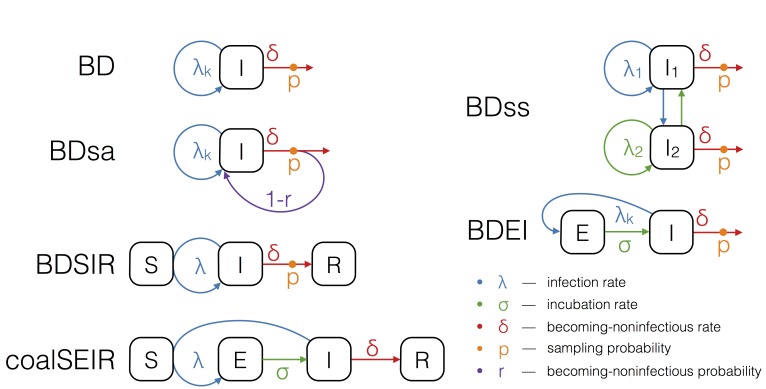
The boxes S, E, I and R refer to the host compartments for susceptible, exposed, infected and recovered/removed individuals, respectively. Rates with subscript k can change over time in a piecewise constant fashion.

The larger outbreak, consisting of 72 Ebola sequences, is analysed in BEAST2^13^ to estimate the epidemiological parameters relevant to the epidemic. We employ birth-death and coalescent approaches as models for epidemic spread.

Birth-death models assume a transmission rate with which infected individuals transmit, a becoming-noninfectious rate with which infected individuals recover or die, and a sampling probability, which is the probability at which an infectious person is sampled and sequenced. Such a model naturally accounts for incomplete sampling and, since the sampling probability is a parameter in our model, this quantity may also be estimated. In particular, we run birth-death analyses using the models depicted in Figure 1. We explain the assumptions of these models in the following.

The birth-death (BD) model^17^ allows the three parameters, transmission rate, becoming-noninfectious rate, and sampling probability to change in a piecewise constant fashion.

To model the spread of EBOV more realistically, we further extend the birth-death model to allow for an exposed class of infected people. The exposed class is entered upon infection, and an exposed individual moves from the exposed to the infectious class with a constant incubation rate. This model is referred to as the birth-death exposed-infected (BDEI) model^18^^,^ 19. In the BDEI model we assume that only infectious people are sampled, since exposed patients are asymptomatic.

BD and BDEI assume that individuals become noninfectious upon sampling. As Ebola may be transmitted also after sampling (transmission at funerals constitutes a major source of infection^2^^,^ 3 ^,^ 20) we further run the birth-death sampled-ancestors (BDsa) model^21^, which extends BD by assuming that sampled individuals become noninfectious upon sampling with probability *r* and remain infectious with probability 1-*r*. When *r*<1 the phylogeny may contain sampled ancestors, meaning samples do not have to coincide with tips in the tree, but a sample in the tree may have sampled descendants.

The BDSIR model^22^ is a variant of the BD model in which we explicitly account for susceptible hosts, meaning the epidemic slows down once the number of susceptible hosts declines. This model includes an explicit susceptible class and the number of initial susceptible hosts as a parameter, which was estimated using a LogNormal(8,4) prior distribution.

We also fit a deterministic coalescent model to the EBOV sequence data. We use the structured coalescent framework of Volz (2012)^15^, assuming an exposed and infectious class (as in the BDEI model), to probabilistically take into account whether lineages reside in exposed or infectious individuals. This coalescent SEIR model (coalSEIR) was implemented in BEAST2 and epidemiological parameters were estimated along with the genealogy from the sequence data, with the initial number of susceptible hosts set to 1 million, following Althaus (2014)^23^.

In all analyses we first assumed a constant basic reproductive number R_0_, which is the ratio of the transmission rate over the becoming-noninfectious rate. Second we allowed the reproductive number to change twice: at the time of the oldest sample (May 26) and midway between the oldest and youngest samples (June 6). The becoming-noninfectious rate and the sampling probability were assumed to remain constant throughout the epidemic outbreak.

We assumed the following Bayesian prior distributions for our analyses. The prior for R_0_ is LogNormal(0,1.25). The time of origin, i.e. the time of infection of the first person in the Sierra Leone outbreak, was assumed to be uniform during the 6 (and for computational reasons in some analyses, 3) months prior to the most recent sample at time 18 June 2014, thus any start time of the Sierra Leone outbreak from 18 December 2014 (or 18 March 2014) was equally likely. For the incubation rate and the becoming-noninfectious rate we assumed a Gamma prior with shape 0.5 and scale 1/6 days^-1^, truncated, such that the periods of being exposed and infectious lie between 1 and 26 days, and such that all times in this interval have considerable support. The median of these priors is 0.11 days^-1^, meaning that the expected time of being exposed and infectious is 9 days each. As no sequencing effort has been performed prior to the oldest sample, collected on 25 May 2014, we assume that the sampling probability is 0 prior to that date and constant afterwards. After that date, we assume a uniform prior on [0,1] for the sampling probability in the analyses without exposed class. To improve computational performance in the more complex BDEI model, we assume a Beta(70,30) prior distribution, supporting a sampling proportion around 70%, based on our own results as well as Gire *et al.* (2014)^12^ , and also fix the mean clock rate to 1.984e^-3^/site/year^12^ . The priors on all epidemiological parameters as well as the mean clock rate were identical between the coalSEIR and BDEI models.

Instead of reporting the becoming-noninfectious rate and the incubation rate, we report their inverse values, which are the expected times of being exposed (incubation time) and being infectious. We report the median posterior value for each parameter together with the shortest interval containing 95% of the posterior samples.

**Maximum likelihood analysis using birth-death models**

**As a comparison, we performed maximum likelihood (ML) parameter estimation using the posterior trees from Gire *et al.* (2014)^12^. Again, we first eliminated the Guinea samples and the 6 samples from the second Sierra Leone outbreak. Thus all trees analyzed consist of 72 tips. From the 10001 posterior trees provided by the authors of Gire *et al. *(2014)^12^ , we eliminated the first 1001 trees as burn-in, and then chose every 100th tree from the remaining 9000 trees, yielding a set of 90 trees. For these 90 trees, we performed an analysis under the BD model with constant and time-varying reproductive number and BDEI with constant R_0_ using the R package TreePar v3.1^16^. Additionally, we applied a birth-death model to the trees quantifying the amount of superspreading in the population, BDss^18^. This model extends the constant-rate BD model, assuming that individuals belong to either one of two classes with a unique R_0_. Individuals transmit to both classes. We report the median maximum likelihood value together with the shortest interval containing 95% of the ML estimates from all 90 trees. **

## Epidemiological parameter estimates

**95% HPD intervals of estimated parameters for different phylodynamic methods:**
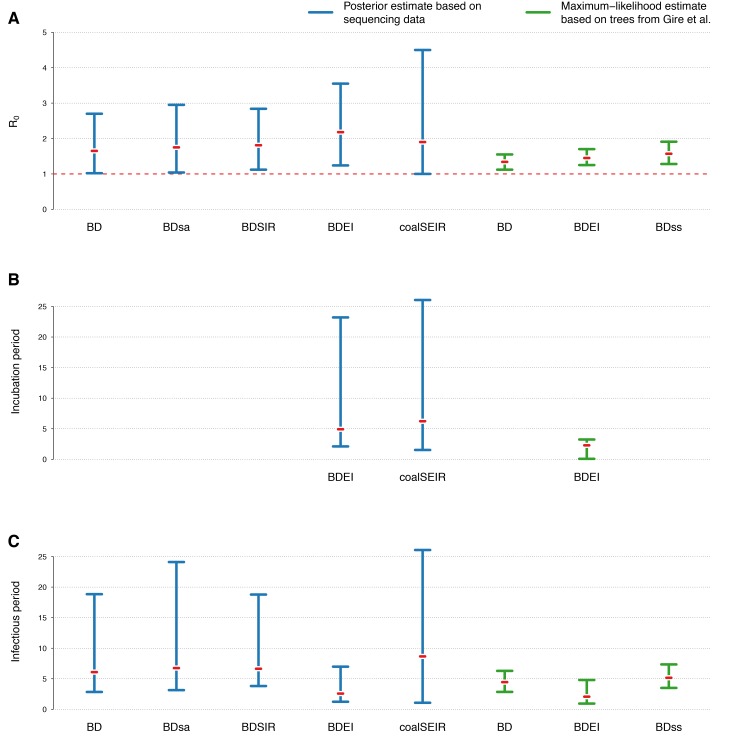
(A) R_0_, (B) incubation period and (C) infectious period. The estimates from methods allowing the reproductive number to change over time are omitted. We also omit estimates from models where we fixed any of the other model parameters (except for the maximum-likelihood estimates, where the sampling probability is always fixed).

Figure 2 displays the estimated R_0_ values for the different phylodynamic methods. Overall the different Bayesian methods simultaneously inferring trees and parameters yield median estimates between 1.65-2.18. The maximum likelihood methods inferring parameters based on fixed trees obtain lower estimates. In the following we discuss the results in detail.

**Bayesian birth-death analysis**

**Table 1: Posterior birth-death parameter estimates based on sequencing data.** Models followed by 1 estimate only a constant R_0_, while models followed by 3 allow the reproductive number to change 3 times over the course of the epidemic. ^*^Sampling probability prior: Beta(70,30), mean clock rate fixed to 1.984e-3 [Gire 2014], origin prior: Unif(0,3months)

	R_0_/R_e __initial _	R_e_ _middle_	R_e_ _recent _	Incubation time (days)	Infectious time (days)	Sampling probability	Epidemic origin	Tree MRCA
BD 1	1.65 _(1.02-2.70)_	-	-	-	6.09 _(2.84-18.84)_	0.65 _(0.20-1.00)_	May 7 _(7/4-22/5)_	May 15 _(3/5-22/5)_
BD 3	0.95 _(0.22-2.56)_	1.57 _(0.73-2.91)_	1.81 _(1.07-3.03)_	-	6.15 _(3.22-17.94)_	0.70 _(0.27-1.00)_	April 8 _(30/12-21/5)_	May 12 _(24/4-23/5)_
BDsa 1	1.75 _(1.04-2.95)_	-	-	-	6.75 _(3.14-24.10)_	0.60 _(0.17-1.00)_	May 8 _(10/4-22/5)_	May 15 _(3/5-23/5)_
BDsa 3	0.96 _(0.20-2.65)_	1.61 _(0.74-3.00) _	1.88 _(1.09-3.23) _	-	6.54 _(3.24-22.10)_	0.65 _(0.19-1.00)_	April 9 _(31/12-20/5)_	May 12 _(24/4-23/5)_
BDSIR	1.81 _(1.12-2.84)_	-	-	-	6.64 _(3.61-18.78)_	0.70 _(0.24-1.00)_	May 4 _(11/4-19/5)_	May 15 _(3/5-22/5)_
BDEI 1^*^	2.18 _(1.46-3.22)_	-	-	*5.6 * *_(fixed)_*	2.29 _(1.23-5.62)_	0.72 _(0.63-0.80)_	May 10 _(13/4-23/5)_	May 14 _(3/5-22/5)_
BDEI 3^*^	1.77 _(0.59-4.35)_	1.92 _(0.80-3.64)_	2.86 _(1.58-4.78)_	*5.6 * *_(fixed)_*	2.75 _(1.41-7.07)_	0.71 _(0.62-0.79)_	May 8 _(14/3-22/5)_	May 13 _(28/4-22/5)_
BDEI 1^*^	1.85 _(1.17-2.76)_	-	-	*2.3 * *_(fixed)_*	3.92 _(2.15-9.47)_	0.71 _(0.62-0.79)_	May 9 _(15/4-21/5)_	May 14 _(4/5-22/5)_
BDEI 3^*^	1.63 _(0.54-4.09)_	1.66 _(0.71-3.13)_	2.45 _(1.28-4.17)_	*2.3 * *_(fixed)_*	4.72 _(2.46-10.74)_	0.71 _(0.62-0.79)_	May 5 _(12/3-23/5)_	May 13 _(29/4-22/5)_
BDEI 1^*^	**2.18 ** **_(1.24-3.55)_**	**-**	**-**	**4.92 ** **_(2.11-23.20)_**	**2.58 ** **_(1.24-6.98)_**	**0.71 ** **_(0.62-0.80)_**	**May 8** **_(10/4-21/5)_**	**May 14** **_(3/5-22/5)_**
BDEI 3^*^	2.00 _(0.66-5.46)_	1.85 _(0.57-3.71)_	3.15 _(1.43-6.09)_	5.92 _(2.49-24.92)_	2.71 _(1.28-9.22)_	0.71 _(0.63-0.80)_	May 5 _(3/4-21/5)_	May 13 _(30/4-22/5)_

Table 1 shows the results of the Bayesian birth-death analyses, including the times of origin and of the most recent common ancestor (MRCA). Under the constant birth-death-sampling model (BD1), we estimate an R_0_ of 1.65 (1.02-2.70), a sampling proportion of 65% (20-100%) and an infectious period of 6 days (2.84-18.84). There is no indication of a change in the reproductive number before mid June.

Since the BD model does not account for an incubation period, we also perform a simulation study in which we simulate an outbreak with incubation periods and analyse it under BD. This simulation shows that we can robustly estimate R_0 _under the BD model even without including an explicit incubation period, and that the estimate of the infectious period is roughly equal to the sum of incubation and infectious period in the simulations (Supplementary Table 1).

Allowing individuals to stay infectious upon sampling using the sampled ancestors model (BDsa) leads to very similar estimates of the epidemiological parameters. In fact, we only estimate two sampled ancestors in our dataset and the probability to become noninfectious upon sampling is large, 0.93 (0.71-1.00).

The epidemiological parameters are also estimated similarly under the BDSIR model, in which incidence can decline over time due to depletion of susceptible hosts. The initial number of susceptible individuals is estimated at 46000 (median) with large uncertainty (95% HPD, 380-534000). Estimating a similar R_0_ under a model that explicitly allows for the depletion of susceptible hosts over time suggests that the epidemic had not surpassed the exponential growth phase by mid June.

Using the BDEI model, which takes the incubation period into account, leads to slightly larger estimates of the basic reproductive number, 2.18 (1.24-3.55). There is a lot of uncertainty in our estimate of the incubation period of 5 days (2.11-23.20 days). Figure 3B shows that there is only little deviation of the posterior from the prior. The infectious period is estimated to be rather short, 2.58 days (1.24-6.98). Here, the posterior deviates a lot from the prior (Figure 3C). When we fix the incubation time to a shorter (2.3 days) or longer (5.6 days, as in Althaus (2014)^23^) period, we see a slight decrease or increase in the basic reproductive number, respectively. The times of origin (median May 8) and the MRCA (median May 14) show little variation.

**Bayesian analysis in a coalescent framework**

**Table 2: Posterior coalescent parameter estimates based on sequencing data.** 

	R_0_	Incubation time (days)	Infectious time (days)	Epidemic origin	Tree MRCA
coalSEIR	1.90 _(1.00-4.50)_	6.23 _(1.53-26.05)_	8.66 _(1.076-26.07)_	May 5 _(24/3-20/5)_	May 14 _(29/4-22/5)_

**Prior and posterior distributions of estimated parameters.**
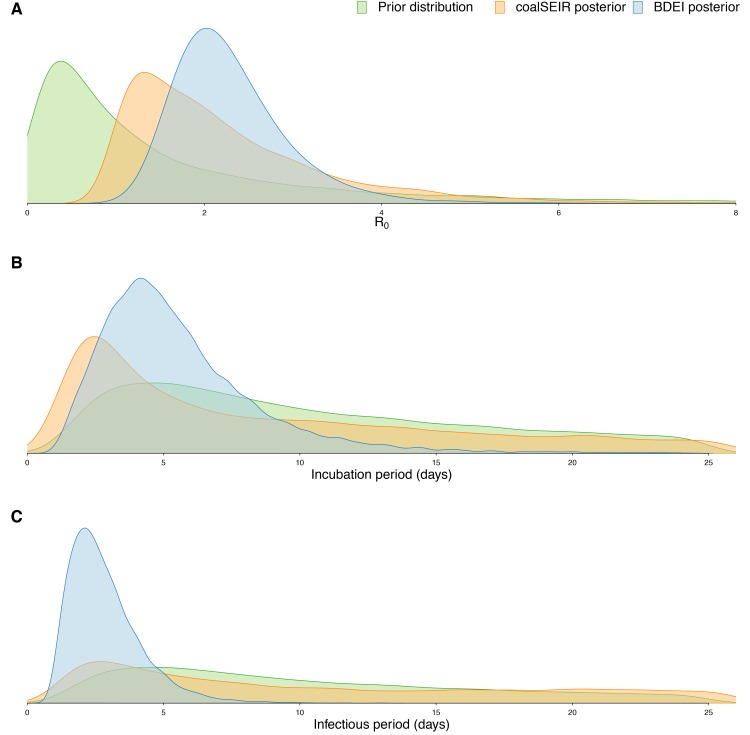
(A) R_0_, (B) incubation period and (C) infectious period. We only show the posterior distributions for coalSEIR and BDEI with a constant reproductive number.

Epidemiological estimates obtained under the coalSEIR model were generally very similar to those obtained under the BDEI model, which was expected given that both approaches include an incubation period and account for uncertainty in the genealogy. Table 2 shows the estimated medians and 95% HPD intervals for the coalSEIR model parameters. While the credible intervals for R_0 _were wider under the coalSEIR than for the BDEI, R_0 _was estimated to be 1.90, just lower than under the BDEI model. Likewise, both methods returned a median epidemic origin time in the first weeks of May. We are not able to precisely estimate the duration of the exposed or infectious periods under the coalescent model, and our estimates appear to be largely informed by the prior, see Figure 3B and C.

**Maximum Likelihood birth-death analyses based on fixed trees**

**Table 3: Maximum-likelihood birth-death parameter estimates based on trees from Gire et al. (2014):** 

	R_0_/R_e_ _initial_	R_e_ _middle_	R_e_ _recent_	Incubation time (days)	Infectious time (days)	Sampling probability
BD 1	1.34 _(1.12-1.55)_	-	-	-	4.45 _(2.85-6.29)_	*0.7 * *_(fixed)_*
BD 3	1.18 _(0.54-1.72)_	1.17 _(0.87-1.59)_	1.62 _(1.37-1.90)_	-	4.74 _(3.26-6.99)_	*0.7 * *_(fixed)_*
**BDEI 1**	**1.45 ** **_(1.25-1.70)_**	**-**	**-**	**2.29 ** **_(0.08-3.24)_**	**2.07 ** **_(0.94-4.80)_**	***0.7 *** ***_(fixed)_***
BD 1	1.24 _(1.08-1.37)_	-	-	-	3.04 _(2.16-4.32)_	*0.35 * *_(fixed)_*
BD 3	1.02 _(0.63-1.50)_	1.10 _(0.87-1.47)_	1.44 _(1.29-1.69)_	-	3.28 _(2.26-4.60)_	*0.35 * *_(fixed)_*
BDEI 1	1.31 _(1.19-1.45)_	-	-	1.81 _(1.22-2.58)_	1.22 _(0.61-2.22)_	*0.35 * *_(fixed)_*

**Table 4: Maximum-likelihood birth-death parameter estimates based on trees from Gire et al. (2014) under a superspreader model.** 

	R_0_ _(overall)_	R_0_ _(class 1)_	R_0_ _(class 2)_	Fraction class 1	Infectious time (days)	Sampling probability
BDss	1.57 _(1.28-1.91)_	2.63 _(1.42-8.31)_	0.84 _(0.00-1.40)_	0.45 _(0.07-0.87)_	5.16 _(3.50-7.35)_	*0.7 * *_(fixed)_*

Finally, we performed maximum likelihood parameter inference on fixed trees from Gire *et al.* (2014)^12^ . Because not all four parameters are jointly identifiable^24^, and because our Bayesian analysis confirmed previous estimates of the sampling probability, we fixed this parameter to 0.7 for times more recent than the oldest sample. Again, sampling probability was set to 0 prior to the oldest sample. To understand the sensitivity of our estimates with respect to this setting, we performed a second analysis fixing the sampling probability to 0.35.

For each of the 90 posterior trees, we obtained the maximum likelihood parameter estimates, see Table 3. Overall, assuming different fixed sampling probabilities did not significantly affect estimates. R_0_ was estimated slightly lower compared to the full Bayesian analyses above (medians 1.31-1.45). Again we did not find support for the reproductive number changing through time. A likelihood ratio test, comparing the results for three intervals for the reproductive number vs. a constant R_0,_ does not support three intervals for the effective reproductive number over one interval (for a sampling probability of 0.7, 9 trees out of 90 supported three intervals for R_e_ at the 95% level, and for a sampling probability of 0.35, 11 trees supported three intervals).

The upper bound for the number of days in the infected class across all analyses is 6.99 days. Thus, both full Bayesian and maximum likelihood methods suggest a time in exposed and infectious class that is lower than previous estimates.

As in the Bayesian analyses, when applying the BDEI method to the 90 Sierra Leone Ebola trees we obtain a slightly higher R_0_ when including the incubation period into the model.

When applying a birth-death model assuming two population groups with unique transmission rates, we observe that half of the population appears to have a large R_0_ (median 2.63, 95% HPD 1.42-8.31), and the other half does not appear to effectively spread the disease (R_0_ median 0.84, 95% HPD 0.00-1.40). However, likelihood ratio tests do not strongly support the structured model over the unstructured model.

## Discussion

We used phylodynamic methods to estimate key epidemiological parameters of the current West African EBOV outbreak in Sierra Leone from sequencing data. Although we used a wide range of different models, we consistently recovered very similar estimates. In particular, we estimated the basic reproductive number of EBOV in Sierra Leone up to the time of the most recent sample (18 June 2014). The medians across the Bayesian methods were 1.65-2.18, with the most plausible model (BDEI) yielding a median estimate of 2.18 (95% HPD 1.24-3.55). We did not find any support for a reduction of the reproductive number prior to the most recent sample. Thus our results show that public health interventions during May and June were likely ineffective at reducing transmission in Sierra Leone. Furthermore, analyses suggest that there might be superspreaders among the infected population, however the significance of the population structure results should be reevaluated once larger datasets are available. We estimate expected incubation and infectious periods of 4.92 (2.11-23.20) and 2.58 (1.24-6.98) days. Using our birth-death methods, we confirm the previously estimated sampling proportion of 70%.

Our R_0_ estimates are within the range of estimates for previous outbreaks and other estimates for the current epidemic. For the 1995 EBOV Kikwit outbreak in the Democratic Republic of the Congo, R_0 _was estimated as 1.359±0.128^25^, 1.83±0.06^26^ or 2.7 (1.9-2.8)^20^. Towers *et al. *(2014)^27^ estimate an R_0_ of about 1.5 for the current West African EBOV epidemic, but only R_0_=1.2 (1.0,1.5) for the Sierra Leone epidemic, assuming incubation and infectious time periods of at most 7 days. Gomes *et al.* (2014)^28^ estimate an R_0_ of 1.8 (1.5-2.0) for the current West African EBOV outbreak while Althaus (2014)^23^ estimates an R_0_ of 2.53 (2.41-2.67) for the epidemic in Sierra Leone. Althaus (2014)^23^ further provides estimates of R_0_ for Guinea, 1.51 (1.50-1.52), and Liberia, 1.59 (1.57-1.60). Moreover he estimates that the R_e_ in Sierra Leone has been declining since the onset of control measures and dropped below 1 during July. During the period from our samples his estimates of R_e_ vary between 2.7 and 1.47. Nishiura and Chowell (2014)^29^ give estimates of R_e_ in Sierra Leone and Liberia of between 1.4 and 1.7 during June and July, with R_e_ in Guinea fluctuating erratically around 1 during the same period. Fisman *et al. *(2014)^30^ estimates values of R_0 _between 1.66 and 2.19 for the West African epidemic, however they estimate an R_0_ of 8.33 for the epidemic in Sierra Leone alone which is clearly outside our HPD intervals. The WHO Ebola Response Team estimated an R_0_ of 2.02 (1.79-2.26) for Sierra Leone from empirical data^32^. They also provide estimates for Guinea, 1.71 (1.44-2.01), and Liberia, 1.83 (1.72-1.94). These estimates are consistent with our estimates of R_0_.

We estimate short incubation and infectious periods with all of our birth-death methods. Estimates of the exposed and infectious periods for the 1995 EBOV Kikwit outbreak range from 5.3±0.23 and 5.61±0.19 days, respectively^26^, to 10.11±0.713 and 6.52±0.56 days^25^. However, for the 2000 Sudan Ebola virus (SUDV) outbreak in Uganda, Chowell *et al.* (2004)^26^ estimated the exposed and infectious periods to be 3.35±0.19 and 3.5±0.67 days. To the best of our knowledge the only estimates of the exposed and infectious periods for the current West African EBOV epidemic are by the WHO Ebola Response Team^32^, based on observational data. They estimate an incubation period of 9.0±8.1 days (median of 8 days) for 201 patients in Sierra Leone with single exposures. No overall infectious period is estimated, but instead the authors provide separate estimates for the infectious period based on disease outcome. From the onset of symptoms in patients sampled in Sierra Leone they estimate a period of 8.6±6.9 days (from 128 patients) until death, 17.2±6.2 days (from 70 patients) until hospital discharge and 4.6±5.1 days (from 395 patients) until hospitalisation.

Our HPD interval estimates for the incubation period are in line with other estimates, however our estimates for the infectious period are substantially shorter than estimates from the current epidemic^32^. Judging by the amount of variation in both the estimates from observational and genetic data we conclude that the incubation and infectious periods are highly variable and difficult to estimate accurately. However, we recover consistent estimates for the total time of infection (incubation + infectious periods), meaning there is a significant amount of information in the present dataset on the length of the infection. Sequencing data from more patients might help to get more confined credible intervals.

We see that a method accounting for an incubation period yields higher R_0_ estimates compared to a method assuming all infected individuals are infectious. As having an exposed class/incubation period will slow the initial growth of the epidemic, it is likely that estimates obtained under models that do not include the incubation period are lower to compensate for the slower growth rate. Thus it makes sense that R_0_ was estimated to be higher when the incubation period is included.

It is also noteworthy that both our BDEI and coalSEIR analyses converged on similar estimates for R_0_ and the epidemic origin. Thus our epidemiological estimates appear robust to the specific assumptions of these two models. Nonetheless, we do observe that our credible intervals for R_0_ and the exposed and infectious periods are considerably wider under the coalescent than the birth-death model. This may seem counterintuitive as the deterministic coalescent models used here ignore demographic stochasticity and should therefore underestimate the true level of uncertainty about the parameters. The confidence intervals being in fact wider under the coalescent may reflect the fact that the birth-death models are using information entering from the sampling times (while the coalescent conditions on sampling) to obtain more precise estimates of the epidemiological parameters.

Overall, we show that our inferences of the epidemiological dynamics of the current West African EBOV outbreak are robust to the model used and also consistent with estimates from previous outbreaks as well as other estimates of the current epidemic. Our hope is that more sequencing data from the epidemic will be made available in the immediate future. New data will allow us to estimate how the effective reproductive number has changed since June and allow us to estimate the incubation and infectious periods more reliably. Such estimates would be invaluable not only for evaluating the success of containment efforts, but also for planning future interventions.

## Software availability

All Bayesian methods will become available within the BEAST2^13^ add-on “phylodynamics” (https://github.com/BEAST2-Dev/phylodynamics) and are available directly from us prior to the official release. The maximum likelihood methods are available within our R packages TreeSim v2.1^31^ and TreePar v3.1^16^. We provide an R script specific to the Ebola analyses on our website (www.bsse.ethz.ch/cevo).

## Competing Interests

The authors have declared that no competing interests exist.

## Authorship

1. T.S. and D.K. contributed equally to this work.
2. L.d.P. and T.S. designed the study.
3. T.S., D.K. and D.R. performed analyses.
4. All authors analyzed data and all authors contributed to writing the paper.

## Figures and Tables

**(supplement) Prior and posterior distributions of estimated parameters.**
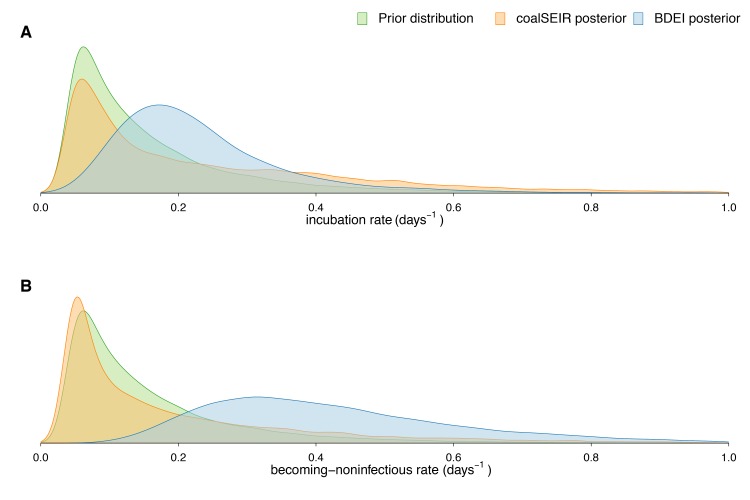
(A) Incubation rate and (B) becoming-noninfectious rate (inverse of time). We only show the posterior distributions for coalSEIR and BDEI with a constant reproductive number.

## Appendix 1

### Supplementary Information

**Supplementary table 1: Posterior birth-death parameter estimates (BD model) for data simulated under BDEI.**

	True value	Median estimate	Relative error	Relative bias	Relative HPD width	95% HPD accuracy
R_0_	1.5	1.561	0.136	0.041	0.764	97%
**Incubation+infectious period**	**11.231**	**14.421**	**0.330**	**0.284**	**1.448**	**100%**
Sampling probability	0.700	0.703	0.076	0.004	1.045	100%
Sampling change time	0.090	0.094	0.467	0.041	3.146	94%
